# The extract of zedoary-turmeric protects rats’ kidneys from damage caused by cisplatin

**DOI:** 10.5455/javar.2025.l950

**Published:** 2025-09-10

**Authors:** Putri Reno Intan, Lisa Andriani Lienggonegoro, Frans Dany, Ariyani Noviantari, Uly Alfi Nikmah, Sukmayati Alegantina, Ratih Rinendyaputri, Ani Isnawati, Sunarno Sunarno, Indah Fajarwati, Sela Septima Mariya, Agus Setiyono, Lina Noviyanti Sutardi, Ekowati Handharyani

**Affiliations:** 1 Animal Biomedical Study Program, IPB Postgraduate School, School of Veterinary Medicine and Biomedical Sciences, IPB University, Bogor, Indonesia; 2 Center for Biomedical Research, Research Organization for Health, National Research and Innovation Agency (BRIN), Cibinong Science Center, Bogor, Indonesia; 3 Research Center for Pharmaceutical Ingredients and Traditional Medicine, Research Organization for Health, National Research and Innovation Agency (BRIN), Cibinong Science Center, Bogor, Indonesia; 4 School of Veterinary Medicine and Biomedical Sciences, IPB University, Bogor, Indonesia; 5 Division of Pharmacy, School of Veterinary Medicine and Biomedical Sciences, IPB University, Bogor, Indonesia

**Keywords:** Acute kidney injury, cisplatin, *Curcuma longa*, inflammation, rat, zedoaria

## Abstract

**Objective:**

The goal of this study was to examine how the combination of *Curcuma zedoaria* (zedoary) and *Curcuma longa* (turmeric) affected things on the kidneys of rats with acute kidney injury (AKI) from cisplatin by assessing the reduction of levels of cysteine-aspartic acid protease 3 (*Caspase-3*), kidney injury molecule-1 (*KIM-1*), and tumor necrosis factor-alpha (*TNF-*α) in renal tissue.

**Materials and Methods:**

There were five groups of rats: a normal control group, a cisplatin control group (CP), and three extract treatment groups (Ext100, Ext200, and Ext400). The CP group got cisplatin on day seven to cause AKI, while the extract group got cisplatin on day seven and the combined extract on days one through nine. On the 10th day, we looked at body weight, kidney weight, histology, and gene expression of *KIM-1*, *TNF-*α, and *Caspase-3* by quantitative real-time polymerase chain reaction. We used SPSS (version 29.0) to do the statistical analyses. We present the data as mean ± SD or SEM and use analysis of variance and Tukey’s post-hoc test to analyze them.

**Results:**

Body weight decreased in the CP group, while it initially decreased and then increased in the extract groups, with Ext200 showing the greatest increase. Histologically, the CP group exhibited severe kidney damage, whereas the Ext200 group showed reduced damage. Gene expression of *Caspase-3*, *KIM-1*, and *TNF-*α was much lower in the Ext100 and Ext200 groups than in the CP group.

**Conclusion:**

Cisplatin-induced AKI caused less damage to the kidneys and less production of *KIM-1*, *TNF-*α, and *Caspase-3*, suggesting that a 200 mg/kg combined extract of Zedoary and Turmeric could be used to prevent or lessen kidney damage. These results show that the extract could preserve the kidneys, which could lead to the creation of other treatments for those who are receiving cisplatin.

## Introduction

Acute kidney injury (AKI) is when the kidneys do not work properly, which can be seen in changes in urine production and high levels of urea and creatinine in the blood [1]. It is linked to the higher death rates, longer hospital stays, and heart problems [2]. AKI is a big problem for people’s health around the world. It affects about 13 million people and kills 1.7 million every year [3,4]. Acute kidney toxicity can arise from various causes, with nephrotoxic injury being a leading factor in acute tubular necrosis [5]. Cisplatin is a nephrotoxic agent that can cause a lot of kidney damage by making it harder for the kidneys to obtain rid of waste and can cause inflammation [6–8]. Cisplatin therapy is a major cause of AKI because it can damage the kidneys even at low doses, and there are currently no effective ways to prevent or treat it [9,10].

AKI can also happen because of some clinical disorders and certain diseases, like cancer. Cancer patients are more likely to suffer AKI because of dehydration, sepsis, and the side effects of cancer treatment, especially cisplatin-based chemotherapy [11]. Cisplatin, a common cancer drug, stops cells from dividing and causes cancer cells to die by making cross-links with deoxyribonucleic acid (DNA) [12]. But it cannot be used in the clinic very often because it has very bad side effects on the kidneys [13]. Cisplatin accumulates in the kidneys during excretion, leading to oxidative stress, inflammation, and proximal tubule injury, which contribute to the development of AKI [14–16].

Various substances have been investigated for their nephroprotective potential in cisplatin-induced AKI. Zedoary and turmeric extracts have shown promising results owing to their bioactive compounds. Curcumin, the main active compound in turmeric, exhibits mitochondrial protective effects, inhibits apoptosis, and suppresses inflammation by blocking proinflammatory transcription factors [11,17]. Similarly, zedoary, closely related to turmeric, exhibits chemoprotective, antimicrobial, and anticancer activities, suggesting its potential as a therapeutic agent for AKI [12].

Although the combined effects of each extract have shown benefits [18,19]. Studies on the combination of zedoary and turmeric in cisplatin-induced nephrotoxicity are required. Further investigation is required to determine their role in mitigating kidney damage and to optimize their therapeutic dosage. Also, there should be a short explanation of the molecular or biochemical pathways of these extracts to illustrate why cysteine-aspartic acid protease 3 (*Caspase-3*), kidney injury molecule-1 (*KIM-1*), and tumor necrosis factor-alpha (*TNF-*α) expression were measured in this study. *TNF-*α is a cytokine that causes inflammation, and *Caspase-3* is a key player in the apoptosis pathway that is triggered by cisplatin treatment. *KIM-1* is a sign that cisplatin is hurting the kidneys. Taking both zedoary and turmeric extracts together is thought to lower the levels of these genes, which should help with inflammation, cell death, and kidney damage caused by cisplatin [12,17]. The goal of this study was to see if combining turmeric (*Curcuma longa*) and zedoary (*Curcuma zedoaria*) extracts could protect the kidneys of rats that had AKI from cisplatin by lowering the levels of *Caspase-3*, *KIM-1*, and *TNF-*α.

## Materials and Methods

### Ethical approval

All experimental procedures involving animals were reviewed and approved by the Ethics Commission for Animal Experimentation of BRIN, Indonesia, in accordance with established guidelines for the care and use of laboratory animals (Approval No. 087/KE.02/SK/05/2023).

### Chemicals and drugs

Cisplatin was obtained from Dankos Farma Limited Company. The drug was supplied in vials, each containing 50 ml of the solution, which included 50 mg of the drug. Cisplatin is a sterile, clear, and colorless solution packed in glass vials at a 50 mg/50 ml vial concentration.

### Extract preparation

Bogor Agricultural University’s Tropical Biopharmacha Research Center in Bogor, West Java, Indonesia, gathered and provided the rhizomes of Turmeric L. and Zedoary (Christm.) Roscoe. For Simplicia, the extraction process involved maceration with pharmaceutical-grade 96% ethanol solvent for three 24 h periods in a row. The sample-to-solvent ratio was 1:10 [20]. A mixture of equal parts of zedoary and turmeric extracts was prepared in a 1:1 ratio. This mixture is referred to as a combined extract in this study.

The extract was initially concentrated before dilution. Normal saline was used as the solvent, and the extract was diluted to final concentrations of 100, 200, and 400 mg/kg BW, respectively. The final volume administered orally to the test animals was adjusted according to their body weights.

### Experimenhtal design

Male *Wistar* rats weighing 170–200 gm at 12 weeks of age were used in this study and acquired from Biofarma Limited Company in Indonesia. All rats were acclimatized to the environmental conditions of the experimental facility. These conditions included a light-dark cycle alternating every 12 h, relative humidity levels between 40% and 70%, and environmental temperatures between 20°C and 25°C. The rats ate a normal diet and could drink as much water as they wanted.

**Figure 1.**
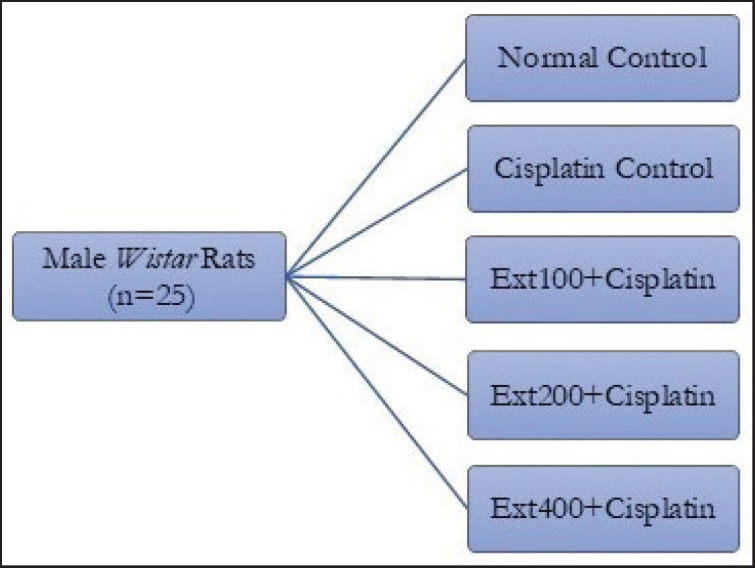
Diagrammatic illustration of the administration of a combined extract of zedoary and turmeric in conjunction with intraperitoneal delivery of cisplatin.

To find out what the extracts did, five groups of five rats each were randomly chosen.The normal control (NC) group was used as a baseline comparison to see how things would have gone without cisplatin-induced AKI. Saline solution was given through the abdomen [intraperitoneal (i.p.)]. The cisplatin control group (CP) group, on the other hand, got cisplatin i.p. to make the AKI model. The Ext100, Ext200, and Ext400 groups were set up to see how combined extracts affected AKI caused by cisplatin (Fig. 1).

The combined extract groups (Ext100, Ext200, and Ext400) were diluted in normal saline and orally administered at 100, 200, and 400 mg/kg. The NC group got one saline injection into the peritoneal cavity on the seventh day of the study. On the other hand, the other groups got 3 mg/kg of cisplatin injected into their peritoneum. The doses of the combined extract and cisplatin were based on the results of previous studies, but with some changes [21,22]. On the 10th day, all of the experimental groups were put to death. The left kidney was evaluated for molecular research, and the right kidney was evaluated for microscopic examination.

### Body and relative kidney weight assessment

Body and relative kidney weights were assessed to evaluate the severity of AKI and the potential protective effects of zedoary and turmeric extracts. Changes in body and kidney weights indicate metabolic stress, inflammation, or toxic accumulation. The rats were weighed on three separate occasions throughout the study: once at the beginning (day 0), again on day 7, and on day 10 (the end of the study). Seventy-two hours after cisplatin injection, a mix of xylazine (10 mg/kg) and ketamine (80 mg/kg) was given directly into the abdomen for anesthesia. Relative kidney weight was calculated by multiplying kidney weight by 100 and dividing the result by the animal’s total weight (in grams) [23].

### Histological examination

Kidney histological examination was performed to assess structural damage caused by cisplatin-induced AKI and the regenerative effects of the treatment, including tubular necrosis, inflammation, and tubular injury, by examination in HE staining [24]. The inspection was conducted using a microscope (EVOS M7000, Thermo, MO, USA) to evaluate histological alterations, and 400× magnification pictures of the renal cortical areas were obtained. A histopathologist inspected the sections under a light microscope, blinded to the therapies administered.

### Molecular analysis

This study looked at the kidneys’ molecules to see how the extract affected cisplatin-induced AKI at *KIM-1*, a key kidney injury biomarker that shows damage to the tubular epithelium, and *Caspase-3* to see how much apoptosis was happening, which showed how much cell death was caused by cisplatin and how well the extract worked, and *TNF-*α levels were assessed to determine the inflammatory response in AKI and the potential anti-inflammatory effects of the zedoary and turmeric extracts.

A homogenizer was used to break up the kidney tissue, and then the manufacturer’s instructions were followed to use the RNA Extraction Kit (Tiangen Biotech, product code: 4992858) to obtain all of the RNA from the kidneys. Using an RT-PCR Kit (Sisco Research Laboratories, 94837), the extracted RNA (100 ng/μl) was turned back into cDNA. KAPA SYBR Fast ROX Low qPCR kit (KK4619) to do quantitative Real-Time PCR (qRT-PCR, Applied Biosystems 7500 Fast instrument) to check the levels of *TNF-*α, *KIM-1*, and *Caspase-3*. The Δcycle threshold approach was used for the data analysis.

The final volume of the PCR mixture was 20 μl. It had 2 µl of cDNA, 100 ng of forward and reverse primers, 10 μl of qPCR, and water that did not contain any nucleases. Before PCR amplification, there were 40 cycles of amplification, denaturation (95°C for 5 sec), and annealing (57°C for 30 sec), followed by an initial denaturation stage (95°C for 3 min). The amplification process was repeated three times for each sample. The relative expression is 2− ΔCt, where ΔCt (Delta Ct) is the difference between the average Ct of the target gene and the average Ct of the reference gene (beta-actin).

Primer sequences for the reference gene *β-actin* and the target genes *TNF-*α, *KIM-1*, and *Caspase-3* (Table 1).

**Table 1.** Primer sequences.

Gene	Sequences		Accession number
*TNF-*α	Forward	TTCGGAACTCACTGGATCCC	NM_012675.3
	Reverse	GGAACAGTCTGGGAAGCTCT	
*KIM-1*	Forward	GTGAGTGGACCAGGCACACA	NM_173149.2
	Reverse	AATCCCTTGATCCATTGTTTTCTT	
*Caspase-3*	Forward	CCGACTTCCTCTATGCTTACTC	NM_012922.2
	Reverse	CGTACAGTTTCAGCATGGC	
*β-actin*	Forward	AGGAGTACGATGAGTCCGGC	NM_031144.3
	Reverse	CGCAGCTCAGTAACAGTCCG	

*KIM-1* = kidney injury molecule-1, qRT-PCR = quantitative real-time polymerase chain reaction, *TNF-*α = tumor necrosis factor alpha.

### Statistical analysis

The average values and SDs show the rats’ kidney and body weights in relation to each other. After a one-way analysis of variance, Tukey’s post-hoc test was used for the assessment. The cutoff for statistical significance was *p* < 0.05. Real-time RNA expression data were obtained by using the mean and the SEM. We used SPSS (version 29.0) on Windows to analyze the data.

## Results and Discussion

### Body and kidney weight changes

**Figure 2.**
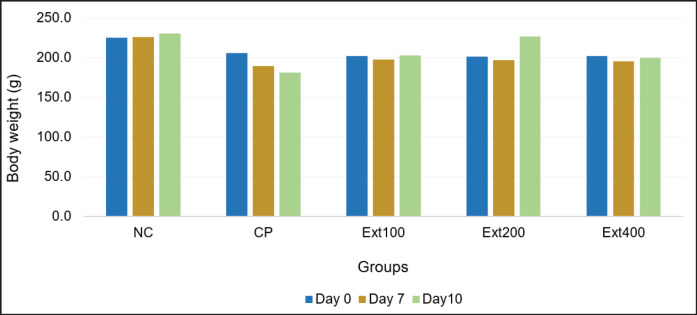
Effect of combined extracts of zedoary and turmeric on body weight measured on days 0, 7, and 10. Ext400 = extract400 + cisplatin, CP = cisplatin control group, Ext100 = extract100 + cisplatin, Ext200 = extract200 + cisplatin, NC = normal control.

Figure 2 shows the body weights of all rats on days 0, 7, and 10. The CP group continuously lost body weight throughout the experiment, whereas the NC group showed slight weight gain. In the treatment groups receiving the combined extract, body weight remained relatively stable; there were no significant differences, except for the Ext200 group, which saw a big rise.

**Figure 3.**
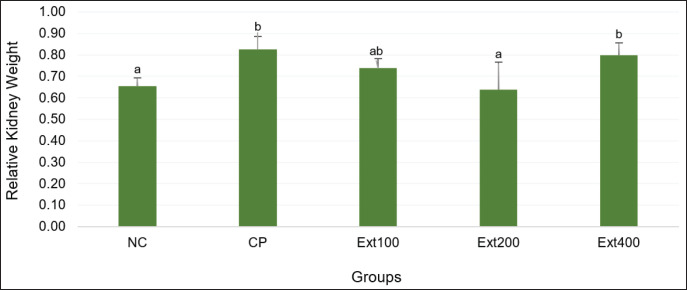
Combined zedoary and turmeric extracts affected relative kidney weight. Data are presented as mean ± SD and were analyzed using one-way analysis of variance and Tukey’s post-hoc tests. Different letters indicate statistically significant differences (*p* < 0.05) between columns. CP = cisplatin control group, Ext100 = extract100 + cisplatin, Ext200 = extract200 + cisplatin, Ext400 = extract400 + cisplatin, *KIM-1* = Kidney Injury Molecule 1, NC = normal control, *TNF-*α = Tumor Necrosis Factor alpha.

Statistical analysis indicated that the CP treatment group lost a lot more weight than the NC group (*p* < 0.05). On the other hand, the extract treatments (Ext100 and Ext400) did not cause any significant weight changes, which means that these doses did not stop cisplatin from causing weight loss. Interestingly, the Ext200 group gained a lot of weight compared to the CP and other extract groups. This evidence suggests that this specific dose might have a protective effect (Table 2). Figure 3 shows that the CP and Ext400 groups had much higher relative kidney weights than the NC group. This is probably because the kidneys were swollen and inflamed. Even though the Ext200 group gained weight, their relative kidney weight stayed about the same as the NC group’s. This evidence suggests that there is a balance between keeping body weight stable and protecting the kidneys.

These results imply that the weight loss caused by cisplatin may be due to increased catabolism and kidney problems; however, the Ext200 dose of the extract seems to work better than other doses to stop such changes from happening. This finding is in line with earlier research that suggested curcumin could help improve metabolic and inflammatory conditions in rats that had been treated with cisplatin [25].

The CP group lost more weight than the NC group in this study. This result is in line with the relative kidney weight measurements, which indicated that the CP group’s kidneys were substantially heavier than the NC group’s. The results of this investigation are similar to those of earlier studies that showed that cisplatin induced a significant decrease in kidney weight compared to the control group [26–28]. The CP group may have lost weight because their catabolism sped up, which might have caused acidosis, loss of appetite, less food intake, or direct injury to the renal tubules, which would have made it harder for them to reabsorb water [29]. When kidney tubules are hurt and cannot reabsorb water properly, the animal may urinate too much and become dehydrated, which could lead to weight loss. Previous research has suggested that inflammation and swelling could be the cause of the rise in kidney weight after cisplatin treatment [29]. Based on previous studies, these clinical indicators were significantly improved by curcumin treatment [25].

**Table 2.** Effect of combined Zedoary and Turmeric extracts on body weight in cisplatin-treated rat.

Groups	Body weight (gm)		Difference
	Day 0	Day 10	(Day 0–Day 10)
NC	225.33 ± 29.55^a^	230.5 ± 29.79^b^	+5.17 ± 5.12^b^
CP	205.67 ±7.06^a^	181.67 ± 8.16^a^	−24.00 ± 6.42^a^
Ext100	202.17 ± 6.88^a^	203.33 ± 10.28^ab^	+1.17 ± 4.17^b^
Ext200	201.16 ± 6.88^a^	227.17 ± 25.70^b^	+26 ± 19.43^c^
Ext400	202.17 ± 3.76^a^	200 ± 8.37^ab^	−2.17 ± 4.92^b^

(+): Increased; (-): The data are displayed using Tukey’s post-hoc tests, one-way analysis of variance, and mean ± SD. Different letters in each column represent statistically significant differences (*p* < 0.05). CP = cisplatin control group, Ext100 = extract100 + cisplatin, Ext200 = extract200 + cisplatin, Ext400 = extract400 + cisplatin, *KIM-1 *= Kidney injury molecule 1, NC = normal control, *TNF-*α = Tumor necrosis factor alpha.

### Histological findings in rats induced by cisplatin

**Figure 4.**
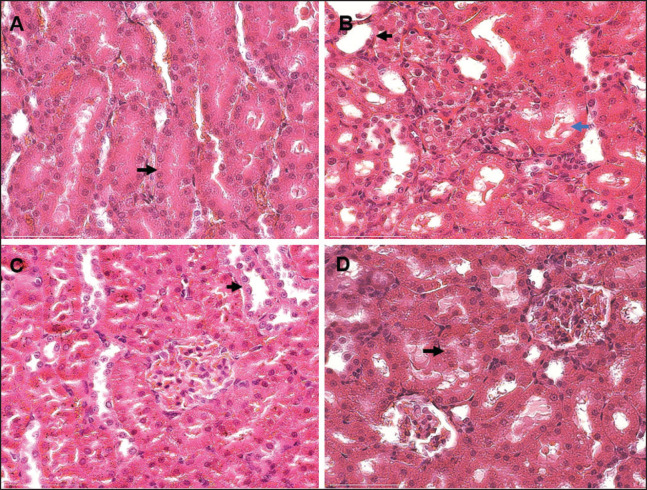
Photomicrographs of kidney sections stained with H&E from rats administered combined extracts of zedoary and turmeric following nephrotoxicity caused by cisplatin. (A). Normal tubule (black arrow); (B) proximal tubule dilatation with epithelial lining atrophy (black arrow) and hyaline cast (blue arrow); (C) tubular degeneration (black arrow); (D) tubular necrosis (black arrow). 75 μ is equal to bars.

Histological analysis revealed normal kidney structure in the NC group. In contrast, the CP group exhibited tubular necrosis in the cortex, proximal tubule dilatation with epithelial lining atrophy, degenerative symptoms in some tubules, and the presence of hyaline casts. At 100 and 200 mg/kg, the combined extract group experienced less tissue damage than the CP group. However, as observed in the CP group, a higher dosage of the combined extract (ext400) caused lesion damage. Microscopic examination confirmed these results, showing that the CP group had more lesions than the Ext200 group. At 200 mg/kg, the combined extract protected against cisplatin-induced kidney damage (Fig. 4). The increased damage observed in the CP group was greater because of the primary mechanism of cisplatin elimination via renal excretion. This process enables the kidneys to accumulate cisplatin at higher concentrations than other organs, leading to nephrotoxicity. Similar histological changes in the kidney, including cisplatin-induced thickening and enlargement of the basement membrane in the proximal tubules of rats, have been previously reported [30,31].

Cisplatin doses that cause kidney damage in mice can be very low (5 mg/kg) or very high (14–18 mg/kg) or even higher (>20 mg/kg) nephrotoxic (10–12 mg/kg). It may be instructive to use a different dose of cisplatin when the time course, severity, and functional, morphological, or molecular changes caused by nephrotoxicity are thoroughly studied. To test possible drugs or treatment plans, though, a strong and validated cisplatin mouse model is needed. Currently, there is no standardized, strong, or validated cisplatin mouse model of AKI that is useful for humans in a clinical or physiological way [32]. When cisplatin builds up in proximal tubular cells, it damages the kidneys by making reactive oxygen species (ROS), which leads to oxidative stress, mitochondrial problems, and the intrinsic caspase pathway, which encourages apoptosis [33].

The combined extract may have a protective effect at lower dosages because it can inhibit inflammatory mediators, such as *TNF-*α, and strengthen antioxidant defense mechanisms, which are essential in AKI pathophysiology.

However, higher dosages of the extract can have contradictory effects, increasing oxidative stress instead of providing protection. Curcumin has anti-inflammatory and antioxidant properties, but a previous study indicated that too much activation of the Nrf2/HO-1 pathway can throw off the redox balance, making kidney damage worse and causing oxidative cytotoxicity [34]. This dose-dependent toxicity explains why higher doses of the extract resulted in increased kidney lesions.

### Messenger ribonucleic acid (mRNA) expression levels using qRT-PCR

**Figure 5.**
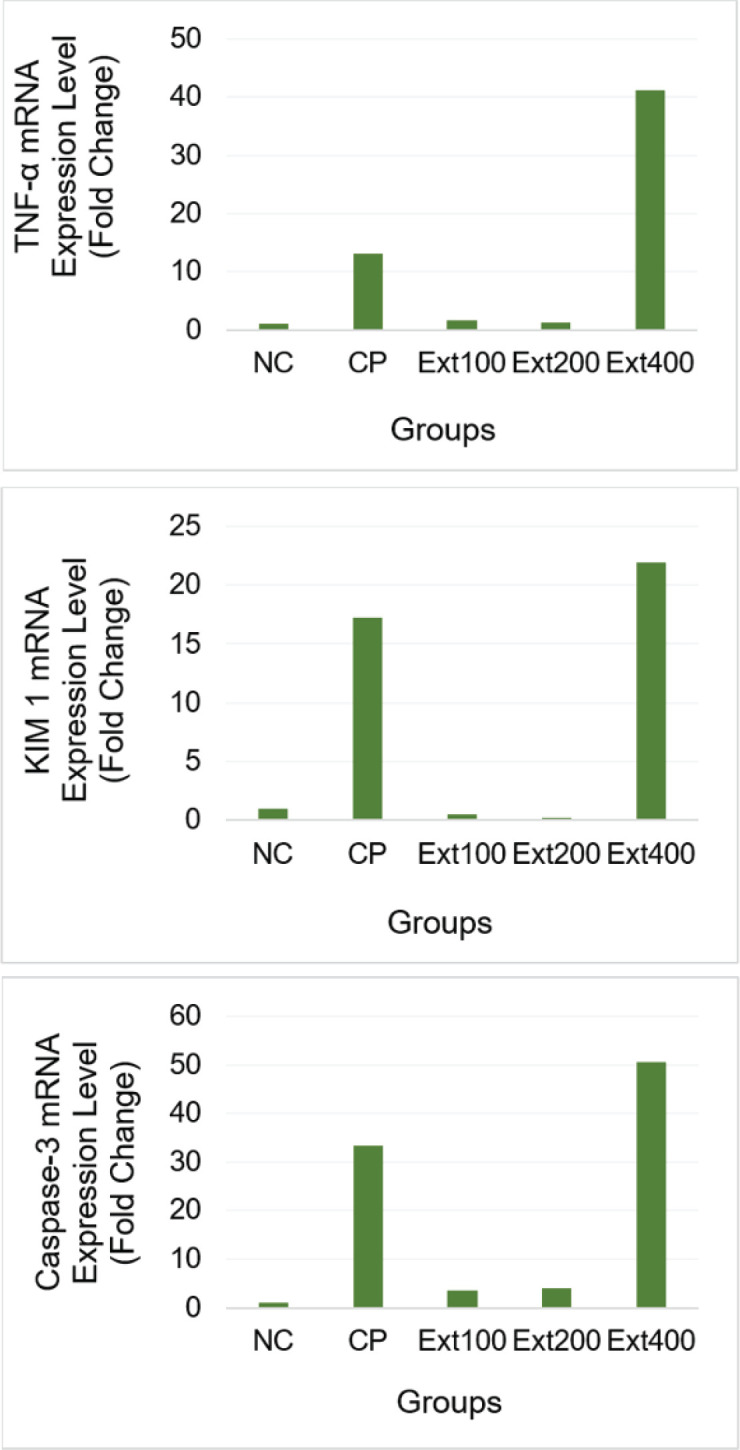
Effects of combined extracts from Zedoary and Turmeric on cisplatin-induced *TNF-*α, *KIM-1*, and *Caspase-3* mRNA levels in rats. Data are presented as mean ± SEM. CP = cisplatin control group, Ext100 = extract100 + cisplatin, Ext200 = extract200 + cisplatin, Ext400 = extract400 + cisplatin, *KIM-1* = Kidney Injury Molecule 1, NC = normal control, *TNF-*α = Tumor Necrosis Factor alpha.

Three days after cisplatin treatment, analysis of kidney tissue samples indicated that the CP and Ext400 groups had substantially higher levels of *Caspase-3* mRNA, *KIM-1*, and *TNF-*α than the NC, Ext100, and Ext200 groups (Fig. 5). These results are consistent with those of other studies showing that rats administered cisplatin had higher levels of *TNF-*α, *KIM-1*, and caspase three gene expression [35,36]. Previous studies have shown that this effect results from frequent interactions between cisplatin and mitochondrial DNA or genomic DNA, which can damage DNA. These interactions halt DNA replication, activate various signaling pathways, and hinder the synthesis of proteins, mRNA, and DNA. Ultimately, these mechanisms result in either necrosis or apoptosis, which kill the cells [25,26,29].

Cisplatin-induced AKI progresses through several inflammatory mechanisms. Both *in vitro* and *in vivo*, cisplatin induces an inflammatory response in kidney tubular cells, characterized by the activation of inflammatory mediators, particularly *TNF-*α [37]. *TNF-*α expression increases in these cells when nuclear factor-kappa B is activated. The link between *TNF-*α and the ensuing cellular damage and death is facilitated by TNF receptors 1 and 2. *TNF-*α levels increased in the CP and Ext400 groups in this study, whereas they decreased in the 100 and 200 mg/kg groups. Owing to its well-known nephrotoxic properties, cisplatin accumulates in the renal tissue at high doses, resulting in tissue necrosis, apoptosis, and inflammation [38,39]. *TNF-*α can promote the synthesis of inflammatory cytokines, such as TGF-β1, and interleukins, such as IL-1, IL-4, and IL-6 [40]. A previous study indicated that curcumin administration before cisplatin injection could restore or maintain low levels of *TNF-*α in the renal tissue [38].

In this study, the extracts were administered seven days before cisplatin injection, indicating that a blend of zedoary and turmeric had a nephroprotective effect. These findings are consistent with another study that found that administering 200 mg/kg of curcumin orally may shield kidney tissue from cisplatin-induced malfunction, as indicated by lower levels of IL-6 and *Caspase-3*. Although the average levels of *TNF-*α and IL-1β decreased, the figures indicated a negligible decrease [41,42]. The observed reduction in *TNF-*α expression may be attributed to bioactive metabolites derived from turmeric rhizome, a plant with several biological properties, such as the ability to reduce inflammation [43].

AKI can be identified by looking for the type 1 transmembrane protein *KIM-1*. It is primarily found in the renal tubules, with higher concentrations in the proximal tubule. This signal indicates damage to the proximal renal tubules and is rapidly expressed in cases of acute nephrotoxic kidney injury [44,45]. Damaged cells make *KIM-1*, which is linked to the regeneration of renal tubular epithelial tissue. It shows up early in kidney disease and is thought to be more accurate than the usual signs of damage to the renal tubules. Therefore, it is commonly employed as a marker of renal impairment after AKI [46,47]. According to these investigations, the CP group’s *KIM-1* expression levels were higher than those of the NC and Ext200 groups. In line with previous studies, a higher level of *KIM-1* in the CP group indicated a higher degree of renal tubule injury [36].

Studies conducted in laboratory settings and animal models have demonstrated that cisplatin triggers renal tubular cell death in a concentration-dependent manner. Several markers indicate apoptotic processes, including caspase activation, DNA degradation, and alterations in cellular morphology. According to previous studies, the prevention of tubular cell death is strongly related to the reduction of cisplatin-induced nephrotoxicity [48–50]. Numerous renoprotective drugs have been shown to reduce tubular apoptosis and partially alleviate AKI. Curcumin reduces apoptosis during cisplatin-induced renal damage, thereby reducing cisplatin-induced renal failure and damage [41,42].

Upregulation of pro-apoptotic proteins, including *Caspase-3* and *TNF-*α, and mitochondrial ROS generation, which promote renal cell death and inflammation, are characteristics of cisplatin nephrotoxicity [33]. This explains why these biochemical markers were more prevalent in the group that received cisplatin treatment.

The levels of *Caspase-3*, *KIM-1*, and *TNF-*α also went up in the high-dose extract group, which could mean that the toxicity is dose-dependent. Too many curcuminoids could make kidney apoptosis worse by messing with mitochondrial activity and generating oxidative stress. Low doses of curcumin protect the kidneys, while excessive doses may harm cells and upset mitochondrial homeostasis [34]. Zedoary and turmeric extracts at doses of 100 and 200 mg/bw have been proven to lower the expression of *Caspase-3* and *TNF-*α levels and *KIM-1*, and this lowers nephrotoxicity. The inhibition of inflammatory pathways is probably what causes this effect [34].

## Conclusion

A nine-day study found that rats administered a 200 mg/kg zedoary-turmeric extract showed reduced *Caspase-3*, *KIM-1*, and *TNF-*α mRNA levels in cisplatin-induced AKI. This was validated by the latest histological study. The 200 mg/kg extract group exhibited fewer tubular lesions than the cisplatin group. These findings suggest the combined extract may protect the kidneys from cisplatin damage. This may improve kidney health in chemotherapy patients. Combined extracts may minimize cisplatin-­induced AKI and offer therapeutic advantages, making them promising candidates for clinical trials. Researchers should study the drug’s effects, determine the best dose, and ensure long-term safety. Clinical trials must determine if it can be utilized as an additional treatment for cisplatin chemotherapy.

## List of abbreviations

BW, Body Weight; *Caspase-3*, cysteine-aspartic acid protease 3; CP, cisplatin control group; delta-Ct, delta cycle threshold; DNA, deoxyribonucleic acid; Ext100, treatment groups with doses of 100 mg/kg and treated with cisplatin; Ext200, treatment groups with doses of 200 mg/kg; Ext400, treatment groups with doses of 400 mg/kg and treated with cisplatin; HE, Hematoxylin and Eosin; i.p., intraperitoneal; *KIM-1*, kidney injury molecule-1; mRNA, messenger ribonucleic acid; NC, normal control; PCR, polymerase chain reaction; qRT-PCR, quantitative real-time polymerase chain reaction; *TNF-*α, tumor necrosis factor-alpha.
